# Equine Rehabilitation: A Scoping Review of the Literature

**DOI:** 10.3390/ani11061508

**Published:** 2021-05-22

**Authors:** Tiago Atalaia, José Prazeres, João Abrantes, Hilary M. Clayton

**Affiliations:** 1 CICANT, MovLab, Lusófona University, Campo Grande 376, 1749-024 Lisbon, Portugal; movlab@ulusofona.pt (T.A.); joao.mcs.abrantes@ulusofona.pt (J.A.); 2 Faculty of Veterinary Medicine, Lusófona University, Campo Grande 376, 1749-024 Lisbon, Portugal; vetstoestevao@gmail.com; 3 College of Veterinary Medicine, Michigan State University, East Lansing, MI 48824, USA

**Keywords:** equine rehabilitation, therapeutic modalities, manual therapy, exercise

## Abstract

**Simple Summary:**

When a horse is diagnosed with a locomotor disorder, the veterinarian treats the specific injury to restore the horse to soundness. Even after the injury has healed, however, the horse may not be fully functional due to persistent limitations in movement or strength in specific areas of the body. As in people, rehabilitation seeks to optimize function and reduce any existing disability using a variety of methods including manual therapy, the use of physical and mechanical agents, and specialized exercise regimes. This study has reviewed the scientific literature with the goal of identifying which types of physical therapy have been described in horses over the past 20 years. The most frequently reported techniques were exercise, electrotherapy, and hydrotherapy but there are relatively few publications describing details of their use and outcomes in clinical cases. This study reviews the methodology and outcomes of rehabilitation in clinical cases. The results highlight the paucity of clinically-based reports on the practical applications of equine rehabilitation and physical therapy.

**Abstract:**

Injuries to the locomotor system are a common problem in athletic horses. Veterinarians address these injuries using appropriate medical, surgical, and pharmacological treatments. During or after recovery from the initial injury, horses may be treated for functional locomotor deficits using specific rehabilitation techniques aimed at restoring full athletic performance. This study reviews the literature to identify which rehabilitative techniques have been used most frequently in horses over the past 20 years, the protocols that were used, and the outcomes of the treatments in naturally occurring injuries and diseases. Publications were identified using keyword selection (Equine Athlete OR Equine OR Horse) AND (Rehabilitation OR Physiotherapy OR Physical Therapy). After removing duplicates and screening papers for suitability, 49 manuscripts were included in the study. The majority of publications that met the inclusion criteria were narrative reviews (49%) in which the authors cited the relatively small number of published evidence-based studies supplemented by personal experience. Observational/descriptive studies were also popular (35%). Randomized control trials accounted for only 10%. The most frequently reported rehabilitation techniques were exercise, electrotherapy, and hydrotherapy. The findings highlight the need for further information regarding type of intervention, parameterization, and outcomes of equine rehabilitation in clinical practice.

## 1. Introduction

Rehabilitation has been defined as the optimization of function and reduction of disability in a patient suffering from a health condition (disease, disorder, injury, or trauma) [1]. Although this definition is based on the human patient, it can be applied equally well to the equine patient and, indeed, to veterinary rehabilitation in general. Thus, equine rehabilitation restores the incapacitated horse to its normal functional capacity and allows the athletic horse to perform at the expected level. Physical therapy employs physical methods to treat pain, disease, or injury by physical means [2]. Physiotherapy is a pseudonym for physical therapy, but since it is a protected term in some countries, this article uses the more general term physical therapy. Physiotherapeutic is the adjective that refers to physical therapy.

Interest in equine rehabilitation and physical therapy is growing rapidly, e.g., [3,4]. As far as the authors are aware, there has not been a published review of the interventions used to rehabilitate horses or the suggested protocols and parameters for application of those interventions. One of the tools used to verify the extent of knowledge in a specific area is a scoping review. A scoping review combines the expertise of the author(s) with various literature searches to address the review topics in a much broader way then a systematic review [5]. Because of this, it serves to identify research gaps and to map the key concepts and main sources of the available evidence [5], and to suggest future research directions in that field.

Thus, the aim of the present study is to address the question, “What are the most frequently used interventions in equine rehabilitation?”. Specifically, we wish to identify which interventions in the area of equine physical therapy and rehabilitation have been most popular in the field over the past 20 years, and to report suggested protocols for their use. 

## 2. Materials and Methods

To answer the research question, a scoping review was conducted following the framework proposed by Arksey and O’Malley [5]. This framework consists of five steps: (1) identify the research question; (2) identify relevant studies; (3) study selection; (4) chart the data; and (5) collate, summarize, and report the data [5]. 

### 2.1. Identifying the Research Question

The proposed research question addresses the need to assess the published veterinary literature in the area of physical therapy and rehabilitation of horses, as a preparatory step towards a larger project being conducted by Lusófona University regarding equine rehabilitation techniques. 

The research question “What are the most frequently used interventions in equine rehabilitation?” defines two main search terms, rehabilitation and horse. Under the term rehabilitation, we searched for studies related to rehabilitation or physiotherapy or physical therapy, which are the terms used most often in this field of research, e.g., [3]. Under the term horse, we included the words horse or equine or equine athlete as being the most frequently used. 

The final keyword selection for the search was (Equine Athlete OR Equine OR Horse) AND (Rehabilitation OR Physiotherapy OR Physical Therapy).

### 2.2. Identifying Relevant Studies

#### 2.2.1. Electronic Databases

The electronic databases used in our search were MEDLINE complete, Cochrane, and Science Direct Elsevier.

#### 2.2.2. Reference Lists

**Figure 1:**
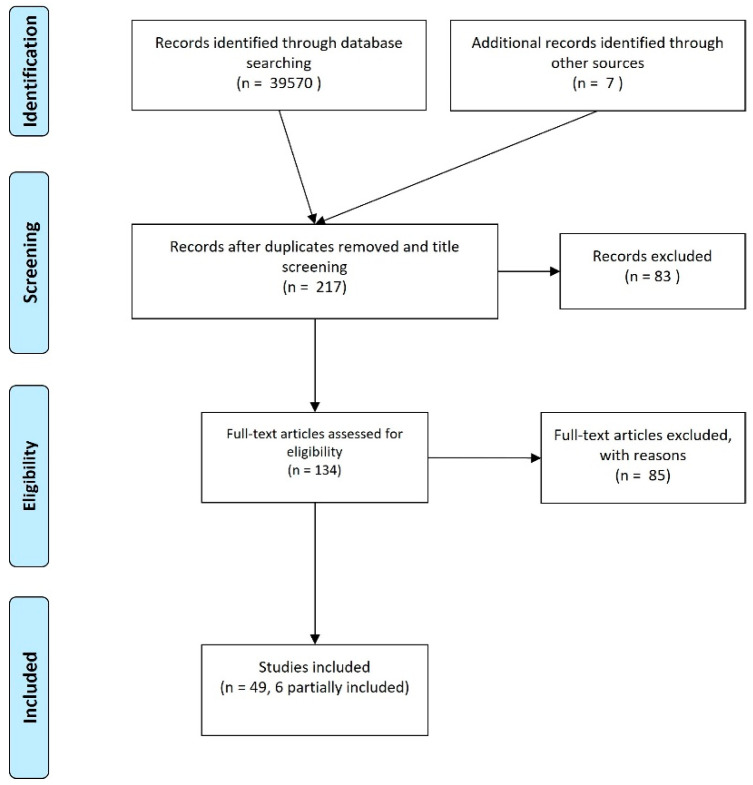
Flowchart of the manuscript retrieval process.

Reference lists were screened to determine whether any manuscript that met the review criteria was missing from the word search. The included manuscripts are indicated in the flowchart (Figure 1).

#### 2.2.3. Manual Search of Other Sources

Some grey literature was screened to check whether important data could be missing from our pool of information. It was also used to understand common word usage in this area as a tool to define keywords.

### 2.3. Study Selection

#### 2.3.1. Inclusion Criteria

The inclusion criteria were all studies (independent of the study type) that address equine rehabilitation from a physical therapy perspective, i.e., using physiotherapeutic methods and modalities for equine rehabilitation.

#### 2.3.2. Exclusion Criteria

All studies addressing other types of intervention (osteopathic, chiropractic, acupuncture, and derivates) were discarded, since the focus was on physical therapy. In addition, studies addressing the benefits of interventions made on sound horses or in vitro models of disease were discarded.

Figure 1 illustrates the manuscript selection process at the different levels. The study selection was performed by three of the authors at different levels of the process. 

A total of 49 studies were selected for inclusion. Six of these presented only partial information, as they were mainly concerned with other intervention methods that were considered as an exclusion criterion. The reasons for exclusion will be explained further in the results and discussion sections.

### 2.4. Charting the Data

After manuscript selection and reading the full text, the final selected manuscripts were charted by key information, including the following:

- Manuscript authors and date
- Type of intervention and comparator (if applicable)
- Populations
- Aims
- Methods

Since we included all types of studies (exploratory, descriptive, clinical trials, etc.), not all the fields were applicable to all the manuscripts. To facilitate the reason why some fields could not be used for a manuscript, we added the “type of study” field in the information collected.

Information screening was conducted by only one of the authors to ensure consistency. 

### 2.5. Collating, Summarizing, and Reporting Data

This phase involved presentation and overview of the review outcome. This is presented in a narrative way according to the key themes identified during the review process, which were related to the interventions used to rehabilitate the equine patient (athlete or non-athlete). Those interventions are divided into manual therapy (including tissue mobilization and joint mobilization techniques), physical agents (including kinesiology taping, thermotherapy, hydrotherapy, and electrotherapy options), and exercise therapy.

## 3. Results and Discussion

The synthesis table is presented in Table 1. The manuscripts that were included were oriented towards rehabilitation from the perspective of physical therapy. However, manuscripts that include complementary approaches in addition to physical therapy as part of the rehabilitation program were considered to contribute to the goal of the present review. The results are described according to the methods of intervention, but first we provide an overview of the types of studies included.

### 3.1. Type of Studies

**Figure 2:**
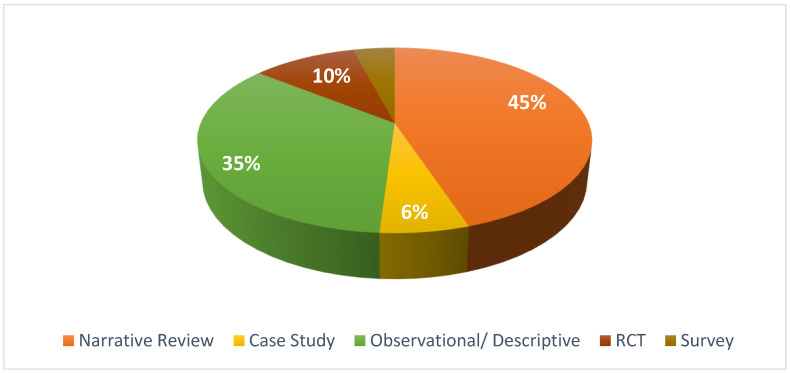
Type of studies included in the data set. RCT: randomized control trial.

From the 49 studies included, the most common type is the narrative review which we defined as a review performed with the aim of describing important themes in equine rehabilitation, but without following a systematic data approach and relying to a large extent on the authors’ experience. The second most frequent is observational/descriptive studies, which describe the effects of a treatment option that was carried out frequently in a veterinary clinic. Some surveys and case reports were also found. The number of clinical trials or randomized clinical trials was very small. Figure 2 shows the distribution of the type of studies in the included manuscripts.

### 3.2. Manual Therapy-Based Interventions

By definition, manual interventions involve applying the hands to the patient’s body for diagnostic or therapeutic purposes. Manual therapy may involve passive stretching, soft tissue mobilization, or joint mobilization to restore the range of motion. As an example of the potential application of manual therapy it has been shown that when sound horses had one fore fetlock joint immobilized in a cast for 7 weeks followed by cast removal and 8 weeks of progressively increasing exercise, the treated fetlock retained 20% reduction in range of motion at the end of the study [54]. Clinical cases involving contracture or limitation of the range of motion after injury or post-surgically may benefit from manual therapy.

#### 3.2.1. Passive Stretching 

Frick [19] reviewed the use of stretching exercises to improve range of motion, prevent injury, and decrease pain. She presented indications and protocols for stretching exercises such as a series of rear limb stretches (hind limb protraction; quadriceps extension—hind limb retraction; hind limb crossover-to stretch adductors; pelvic rocking). It was recommended that each stretch be performed for 3–5 min, once daily, on 3–7 days per week to provide an adequate stimulus. In a review of manual therapies for pain management, Haussler [20] also included a description of the use of stretching for soft tissue restriction and joint stiffness. In a different study, Haussler [35] reviewed joint mobilization and manipulation in the management of the equine athlete and indicated that it was significantly more effective to hold a stretch for 30 s than for 15 s.

#### 3.2.2. Tissue Mobilization

Tissue mobilization includes the techniques of massage, myofascial release, and neural tissue mobilization to break down myofascial adhesions such as scar tissue, to move blood and tissue fluids, and to relax muscle tension and optimize muscle function. The narrative review of Bromiley [6] described massage techniques performed in equine rehabilitation settings, including effleurage, petrissage, tapotement, friction, and skin rolling. Sessions of 20 to 30 min were recommended to treat back pain with the outcome seeming to benefit from the inclusion of passive mobilization. Ridgway and Harman [7] also recommended massage to treat equine back problems but did not define parameters for its use. In the 2006 narrative review of Buchner and Schildboeck [12], none of the cited literature supported a physiological effect associated with general massage techniques but indicated a promising physiological effect in manual lymphatic drainage. On the other hand, Goff [13], stated that massage techniques and neural mobilization were indicated in soft tissue and neural tissue problems, but no parameters were given. 

A narrative review dedicated to massage therapy [16] describes different techniques (effleurage, circular friction, muscle pressure and shaking, skin manipulation, tapotement, petrissage, cross-fiber massage, wringing, classical Swedish techniques, and stroking) as described in the literature, and sometimes associated with stretching, as being beneficial when applied in 10 to 20 min sessions. Haussler [20] described the benefits of massage in alleviating muscle hypertonicity, soft tissue restrictions and pain, and the value of soft tissue mobilization for soft tissue restrictions and pain. A descriptive clinical trial in which the application of effleurage was interspersed by a 3 × 30 s circular kneading for 30 min significantly increased passive and active hind limb protraction [22]. A case study [24] that included massage in a multi-modal physiotherapeutic approach to a case of tetanus reportedly produced a good result but without describing massage parameters. At 6 and 12 months follow up, the foal did not have any deficits. In a survey of rehabilitation modalities used in horse treatments, massage was used by 69% of the respondents [49]. 

#### 3.2.3. Joint Mobilization

Joint mobilization applies a force manually to induce passive physiologic or accessory movements, and active mobilizations of joints. Each joint should be moved in a specific manner, so this technique is best performed by trained professionals. Mobilization techniques include small rhythmic oscillations and gliding movements across the joint directed perpendicular or parallel to the joint’s normal direction of movement to improve motion and normalize joint function with a consequent reduction of stiffness and pain [20]. The first manuscript that refers to this approach is Bromiley [6], in which passive movement is recommended as a good intervention for back problems when associated with massage techniques. Porter [11] recommended 10 repetitions of passive range of motion in the normal physiologic range as a good intervention for joint diseases in horses. Joint mobilization was described as an effective approach for articular, neural, and muscular structures [13]. In rehabilitation of equine articular structures, the recommended techniques were passive mobilization at different amplitudes, velocities, and positions within the available range of motion, integration of both physiological and accessory movements, and the integration of passive accessory mobilization with active movement [13]. 

Haussler [14] indicated the value of joint mobilization in cases of joint stiffness and pain. The same author described joint mobilization and manipulation as important in equine treatment; mobilization was recommended for more generic use, whereas manipulation was more effective in specific conditions [14]. Haussler [35] defined grade 1–2 mobilizations as being characterized by slow oscillations within 25–50% of range of motion and grade 3–4 as being close to the end feel of the joint. A randomized clinical trial using 24 horses was performed to determine the effect of spinal manipulation on vertebral stiffness when added to spinal mobilization [21]. The authors used rhythmic spinal mobilization at five intervertebral sites within the thoracolumbar region. Vertebral stiffness decreased, and there was a further incremental improvement with the addition of spinal manipulation. 

In a case study of a radial fracture, manual passive physiologic mobilization starting 8 weeks post-surgery seemed beneficial, but no treatment parameters were reported [28]. Guedes described manual therapy and movement as elective techniques in painful conditions but did not present any parameters of usage [37]. A survey published in 2018 indicated that range of motion therapies were reportedly used in 71.9% of the responses [49].

### 3.3. Physical and Mechanical Agents

#### 3.3.1. Kinesiological Taping and Bandages

This section includes all interventions that use bandages or taping, with only three manuscripts mentioning these approaches. The first was Goff [13] who added kinesiology tape to manual therapy approaches but did not present any indications for its use. In a research study, kinesiology tape was applied with the FAN technique at 10% tension for 72 h following tibio-patellofemoral joint arthroscopy. Compared with operated but untaped controls, there was a significant reduction in swelling from 24 to 72 h post-surgery [40]. In the survey of Wilson et al. [49], kinesiology taping was part of the intervention in 33% of cases, whereas compression bandages were used in 89.5% of rehabilitation procedures.

#### 3.3.2. Electrotherapy Interventions

According to Bromiley in 1999 [6], common equine electrotherapeutic rehabilitation options were magnetic field therapy (applied with a blanket); transcutaneous electrical nerve stimulation or TENS (once daily for 20–30 min); therapeutic ultrasound (maximal suggested parameters of 1.0 to 0.5 W/cm^2^ with lower powers seeming to be more effective, 3–5 min daily to a maximum of 20 treatment sessions followed by 3 weeks rest); and low-level laser (maximal dosage of 10 J/cm^2^). In a study to assess the efficacy of iontophoresis as a drug delivery option for articular disease, one group received a single intraarticular injection of 4 mL dexamethasone-phosphate (6 mg/mL), and the second group received iontophoretic administration of dexamethasone-phosphate (6 mg/mL) at 4 mA for 40 min in the treated limb and at 0 mA for 40 min in the contralateral (control) limb. Blood and synovial fluid were evaluated but no drug delivery by electrophoresis was detected [8]. 

The benefits of electrical stimulation modalities for rehabilitation of equine joint disease include laser therapy (general report with human-based studies, no parameterization for horses) and therapeutic ultrasound (describes methods, no parameterization recommended) [11]. A 2006 narrative review included information about electrotherapy (TENS) (evidence based on humans and dogs, no parameterization reported), magnetic field therapy (with no conclusive evidence and no parameterization), laser therapy (presenting evidence of the low power option in horses without parameterization), and therapeutic ultrasound (with impressive evidence but no parameterization) [12]. Indications for electrotherapy were presented, including therapeutic ultrasound for which the authors discuss the relative merits of normal to long-wave ultrasound in regard to tissue depth penetration, but without information regarding parameters. This paper also included TENS and iontophoresis, again without suggested parameterization [15]. 

A narrative review described the mechanics of actions, indications, and contraindications for use, and treatment protocols for electrotherapies including neuromuscular electrical stimulation, pulsed electromagnetic field therapy, therapeutic ultrasound, extracorporeal shockwave treatment, laser therapy, and whole-body vibration [36].

In 2018 it was reported that shockwave therapy was part of the treatment in 72.4% of the common procedures, vibration in 39.6%, class 4 laser in 39.9%, therapeutic ultrasound in 39%, class 3 laser in 34.3%, neuromuscular electrical stimulation in 31.8%, TENS in 29.2%, and pulsed electromagnetic field therapy in 22.9% [49]. 

##### Magnetic Field Therapy

In 2017, Guedes described magnetic field therapy as a common option for pain management but without any parameterization [37]. 

Pulsed electromagnetic fields (PEMFs) applied with a blanket were used in a randomized clinical trial in 20 polo ponies with back pain [30]. A placebo blanket was applied to horses in the control group. Using the parameters shown in Table 1, the results failed to indicate significant differences between the PEMF intervention and placebo groups [30].

##### Radial Pressure Wave Therapy

A descriptive study of radial pressure wave therapy applied according to the manufacturer’s recommendations was reported in 65 horses with recurrent proximal suspensory desmitis [10]. Horses received three treatments at 2-week intervals with the parameters shown in Table 1. After the treatments, they performed a controlled exercise program. It was reported that this therapeutic option seemed to provide better results than the placebo [10]. 

##### Extracorporeal Shock Wave Therapy (ESWT)

Parameters for ESWT have been recommended by Kaneps [3] for soft tissue and bone injury (tendinitis, desmitis, osteoarthritis, deep muscle pain), and some proposed protocols were as follows: 

Impulses: small lesions, such as a collateral ligament desmitis of the distal interphalangeal joint, require 1000 impulses per treatment site. Suspensory desmitis lesions most often receive 2000 impulses/treatment. Large areas of the back may require 3000 impulses/treatment.

Energy levels: soft tissue injuries <4 cm deep to the skin: 0.2–0.35 mJ/mm; soft tissue and bone in the heel region: 0.35–0.45 mJ/mm; back disorders: 0.4–0.5 mJ/mm; bucked shins and incomplete fractures: 0.35–0.55 mJ/mm; osteoarthritis: 0.15–0.3 mJ/mm; wounds: 0.1–0.15 mJ/mm.

Focus depth: the focal point for ESWT should be the average depth of the lesion from the skin. Some ESWT devices use gel standoffs to focus the energy at the required depth; others use hand pieces with different focal depths.

For lameness, Contino [42] recommended 2000 pulses to be given with a 12 mm head followed 2 weeks later by 1500 pulses. Compared to the administration of intramuscular polysulfated glycosaminoglycan (PSGAG, 500 mg every 4 days for seven treatments), EWST presented better results and was particularly beneficial for low-motion joints and enthesopathy at the joint capsule insertion [42]. Extracorporeal shockwave therapy was included in the conservative management of foot problems in horses, but without reporting parameterization of usage [43].

A study was designed to assess the effects of ESWT on mechanical nociceptive thresholds and cross-sectional area of multifidus muscle in 12 horses with thoracolumbar pain [53]. The authors reported an increment in the mechanical nociceptive threshold that was more evident in the thoracic region but no significant changes in multifidus cross-sectional area.

##### Therapeutic Ultrasound

A narrative review [3] described the use of therapeutic ultrasound for heat production with a 1 MHz transducer for deeper penetration or 3 MHz for superficial penetration. Energy levels were from 1 to 2 W/cm^2^ applied as a continuous wave for 10 min. Low-intensity ultrasound may be applied once daily for 2 to 3 h in acute injuries and 4 to 6 h in chronic injuries. The device was set at 2.75 MHz at 0.85 W/cm^2^ without the possibility of adjustment. 

Non-invasive, low-frequency ultrasound has been explored for the treatment of lameness, including parameters for its use in acute, subacute, and chronic injuries [50]. Protocols for habituation and treatment were defined according to injury, using different transducers for pulsed and continuous emission, and with different shapes for different wave emission. Recommended protocols for different stages of injury are as follows: 

Acute protocol: week 1: 6 days pulsed emission, flat transducer, 70–80% full power for 10 min; week 2: 6 days pulsed emission, flat transducer, 95% full power for 5 min; weeks 3 and 4, if necessary based on clinical and ultrasound examination, 3 times per week continuous emission, flat transducer, 70–85% full power for 6 min followed by pulsed emission, flat transducer at 80–95% full power for 6 min, followed by pulsed emission, convex transducer at 80–95% full power for 5 min.

Subacute protocol: week 1: 6 days pulsed emission, flat transducer, 70–80% full power for 10 min; week 2: 3 days pulsed emission, flat transducer, 80–95% full power for 6 min followed by pulsed emission, convex focused transducer, 80–95% full power for 5 min; week 3 and 4 (if necessary by clinical assessment), 2 days continuous emission, flat transducer, 70–80% full power for 6 min followed by pulsed emission, flat transducer, 80–95% full power for 6 min followed by pulsed emission, convex focused transducer, 80–95% full power for 5 min.

Chronic protocol: week 1: 6 days continuous emission, flat transducer, 70–80% full power for 6 min followed by pulsed emission, convex transducer, 80–95% full power for 5 min; week 2: 3 days continuous emission, flat transducer, 70–80% full power for 6 min followed by pulsed emission, flat transducer, 80–95% full power for 6 min followed by pulsed emission, convex focused transducer, 80–95% full power for 5 min; weeks 3 and 4 (if needed): 2 days continuous emission, flat transducer, 70–85% full power for 6 min followed by pulsed emission, flat transducer, 80–95% full power for 6 min followed by pulsed emission, convex focused transducer, 80–95% full power for 5 min.

A beneficial result was observed in the cross-sectional area of the suspensory ligament after a mean treatment duration of 3.3 weeks [50]. 

##### Laser Therapy

Proposed parameters for laser therapy include the recommended dosage of 4–12 J/cm^2^ [3]. A study of high-power laser therapy in 150 lame sport horses used the manufacturer’s pre-established protocol for the area of injury [46]. Horses received 250 J/cm^2^ for 20 min daily for 2 consecutive weeks with an exercise rehabilitation protocol and/or pharmacological treatment prescribed at the veterinarian’s discretion. The results suggested a beneficial contribution of high-power laser [46].

##### Whole Body Vibration

A pilot study of the benefits of vibration therapy used a frequency of 50 Hz for 30 min, using a control group with sham procedures, but no differences were found in lameness or gait abnormalities [45]. The use of whole-body vibration for 60 days, five sessions a week for 30 min in horses with chronic lameness, did not produce immediate or long-term benefits [38]. 

#### 3.3.3. Thermal Therapy

Thermal therapy is the application of heat (thermotherapy) or cold (cryotherapy) to the skin to change the temperature of the cutaneous, intra-articular, or other soft tissues as an adjunctive form of therapy for treatment of musculoskeletal and soft tissue injuries. The application of heat may increase skin and joint temperature, improve circulation, relax muscles, and reduce joint stiffness. Additionally, deep heating may decrease the sensitivity of nerves and muscle spindles. Cold applications may reduce pain, decrease swelling, constrict blood vessels, and block nerve impulses. Heat and cold are important treatment methods because they are, for the most part, inexpensive and easy to apply by lay people with only a few precautions. 

The first included manuscript referring to thermotherapy approaches was Ridgway and Harman [7], who described the use of heat and cold therapy for back problems but did not mention any parameters for usage. On the other hand, Porter‘s narrative review [11] described using cold therapy for 30 min several times daily, with more frequent treatments during the first 72 h of the acute phase. Buchner and Schildboeck [12] stated that prolonged or repeated ice water cooling is particularly effective in the equine limbs, while heat affects primarily superficial tissues. No information was provided about parameterization. 

In a narrative review, Paulekas and Haussler [15] indicated cold therapy should be performed for 10–20 min every 2–4 h during the first 48 h post injury and was most effective if applied immediately after injury. They described superficial heat therapy as being beneficial if applied by hot packs at 75° or immersion in hot water. There was no information about water temperature or duration of treatment in the immersion option. In another narrative review [3], some parameterization and usage descriptors were presented, and cryotherapy was stated to be particularly effective in the first 24–48 h after injury for reduction of inflammation and edema and pain control. Ice water immersion, or the application of ice or cold packs for 15 min was recommended. Heat therapy was recommended in chronic injuries when it is usually applied for 20–30 min by warm water irrigation, hot packs, or leg sweats [3]. 

Ice and heat were described as effective methods for pain management, without parameters for their use [37]. A retrospective study of 285 horses described the use of sleeve-style digital cryotherapy to treat distal limb pathological conditions. Three types of application were described: continuous (211 horses), interrupted (57 horses), and intermittent (17 horses) [47]. The cryotherapy was conducted for more than 12 h. The incidence of injury related to this approach was of 7% [47]. Thermal therapy was reported to be used frequently in the 2018 survey [49] in which ice was used in 95.2%, a cold water circulation machine in 48.5%, and heat in 77.6%.

#### 3.3.4. Hydrotherapy

Hydrotherapy uses the physical properties of water, including temperature, pressure, viscosity, and buoyancy for therapeutic purposes that include relieving pain, stimulating blood circulation and treating diseases such as arthritis, muscular diseases, and nerve diseases.

Bromiley [6] referred to hydrotherapy as a common intervention for back problems but did not present parameters of usage. However, swimming is contra-indicated in horses with back pain or diseases due to the fact that they swim with the neck raised and the thoracolumbar spine extended (hollowed) [15]. Porter [11] defined hydrotherapy to include swimming for active exercise and use of an underwater treadmill for active assisted exercises. She recommended a temperature of 62 °C for treating joint diseases but without further information on parameters of usage. Some evidence for the use of hydrotherapy to treat limb injuries has been reported but, again, parameters of use are missing [8]. Swimming for 100–500 m was found useful in the later stages of the rehabilitation program for a radial fracture [28]. 

There have been several surveys and reviews of the use of the water treadmill in which the horse walks or trots on a motorized belt in a chamber filled with water to a chosen level. In 2013, King et al. [29] described the potential value of the water treadmill in rehabilitation of horses with osteoarthritis and secondary musculoskeletal injuries. Published studies of the benefits of water treadmill therapy in osteoarthritic people and dogs were described, and the need for comparable studies in horses was highlighted. Subsequently, this research group published a clinical trial in which 16 horses without clinical abnormalities had bilateral arthroscopies of the middle carpal joints [39]. On one randomly selected side, an osteochondral fragment was created to induce mild osteoarthritis and low-grade lameness. Horses were randomly assigned to treatment and control groups (N = 8). All horses performed treadmill exercise at walk on 5 days/week for 8 weeks beginning on day 15 post-surgically. Initial duration was 5 min/day, increasing by 5 min/week to a maximum of 20 min at walk. Control horses exercised without water in the treadmill, treated horses had water to hip height. Evaluation of forelimb kinematics, symmetry of forelimb loading, activation of select forelimb muscles acting on the carpi, and degree of carpal joint flexion supported the value of the underwater treadmill in rehabilitation of experimentally induced osteoarthritis. 

A review of the use of treadmills in equine rehabilitation [41] presented evidence to support using an overground treadmill to rehabilitate horses with distal limb injuries and back pain. The effects of water treadmill exercise were considered in light of the fact that many horses suffer from multiple musculoskeletal issues, such as lameness and back pain, which may require different approaches. The authors emphasize that successful rehabilitation depends not only on choosing appropriate exercises for the individual case, but also on avoiding inappropriate types of exercise. They list conditions for which exercise on an overground treadmill or a water treadmill are contra-indicated. Guidance is offered regarding the selection of an appropriate depth on the water treadmill. The authors stress the importance of professional monitoring of each horse’s gait pattern during the period of rehabilitation.

A survey published in 2018 investigated why, when, and how the water treadmill is used in equine rehabilitation using three questionnaires that were part of an international survey-based approach [48]. Rehabilitation was reported to account for only 40% of water treadmill use with training being a more popular use (60%). Respondents stressed the importance of adequate habituation to the water treadmill with different centers using an average of 2–3 sessions lasting 10–30 min in water to the depth of the hock or fetlock for this purpose. Significant differences were identified between training and rehabilitation sessions. Training sessions most often used water at hock height with horses walking for 20.5 min and trotting for 8.8 min on average. Water treadmills have been used most frequently in rehabilitation of horses with ligament and tendon injuries. The most common reasons to use the water treadmill for rehabilitation were tendonitis and suspensory desmitis (41%). Water height was most often from just above the fetlock to mid-cannon for rehabilitation sessions. Compared to training, rehabilitation used significantly shallower water, a faster walking speed, and shorter duration (*p* < 0.023 for all three variables). On average the treatment protocol included seven (range 0–14) exercise sessions per week. Therapeutic protocols were similar, but rehabilitation protocols varied significantly between venues [48]. Finally, hydrotherapy was reported to be one of the common interventions in 82.9% of the cases, and the water treadmill was used in 39% [49].

The narrative review of Muñoz et al. [52] describes the application of therapy using the water treadmill in horses with injuries of the superficial or deep digital flexor tendons and their accessory ligaments and in back and joint diseases. The suggestions are backed up by biomechanical information. Control of treadmill speed and water depth are important components of the rehabilitation program. Its use is recommended for treatment of subacute and chronic tendon and ligament injuries and chronic osteoarthritis.

### 3.4. Exercise Therapy

In the past, horses were often rested for a prolonged period of time during rehabilitation, but it is now recognized that it is preferable to return athletic horses to an appropriate type and level of exercise as soon it is safe to do so. Exercise therapy describes the use of specific gaits and movements to enhance the horse’s recovery from injury or disease.

An early reference describing exercise as a therapeutic approach in equine rehabilitation was in Bromiley’s narrative review [6]. The types of exercise described include the treadmill, the horse walker in which horses walk in circles in individual pens that can be controlled for speed and direction, the use of long reins, weighted shoes starting at around 0.25 kg and increasing to around 2.1 kg, for at least 6 weeks, and exercise without the rider’s weight, including on gradients. The author did not present parameters for each type of exercise. 

Another study recommended the Tellington Touch Equine Awareness Movement exercises (TTEAM) that involve touching the horse with a “wand” to stimulate awareness of the body parts, together with negotiating mazes, picking up sticks, and star exercises. It also referred to the importance of cross-training in both training and retraining situations [7]. 

A retrospective study of 17 horses described the conservative management of tibial tuberosity fractures [9]. The progressive exercise program included stall rest for 2 or 3 months with hand-walking starting in the 2nd month and increasing gradually (no amount indicated). Small paddock turn out and walking under saddle (depending on the horse’s attitude) were advised either separately or in combination until the horse was sound at slow trot. The amount of training was then gradually increased [9]. 

In a study about the use of radial pressure wave therapy, a controlled exercise program was recommended. For the first 6 weeks, the horse was on box-rest with daily controlled walking exercise, gradually increasing from 30 to 60 min. Trotting exercise was introduced and gradually increased until week 10–12 [10]. 

In a narrative review about manual therapy in equine treatment programs, Haussler [14] stated the importance of adding stretching exercises (active baited stretches for the axial skeleton, passive stretching for the limbs) with stretches being held for 30 s as an important part of the manual therapy approach. A subsequent narrative review on therapeutic exercise included the use of theraband exercises, induced passive and active cervical bending exercises, spinal reflex movements, axial tail traction, walking through a labyrinth, the star obstacle, walking across elevated bridges, walking over ground poles and raised poles, negotiating raised cavalleti at slow trot, standing on a pedestal, stepping down from a pedestal, walking up and over a pedestal, walking uphill, walking downhill, walking uphill and downhill over poles, and backing uphill. No parameterization was presented [15]. 

Dynamic mobilization exercises or baited stretches involve having the horse follow the path of a treat or a target into specific positions of cervical flexion, extension, or lateral bending. In order to reach the desired positions while remaining balanced, the horse must activate the core musculature. A study of the effects of baited stretches in three neck flexion exercises were studied, namely chin-to-chest, chin-between-carpi, and chin-between-fore fetlocks, in eight riding school horses that were thought to suffer from back pain [17]. The greatest amounts of movement were observed in the most cranial and caudal cervical joints with smaller movements in the mid-cervical region and mid to caudal thoracic regions. A 12-week program based on dynamic mobilization exercises resulted in significant increases in cross-sectional area of the deep spinal stabilizer multifidus muscle on both sides of the spine at five vertebral levels from T10 to L5. Additionally, the muscle cross-sectional areas became more symmetrical on left and right sides [25]. The potential value of dynamic mobilization exercises was explored as a way to increase core strength and vertebral mobilization in horses with back problems [27]. Dynamic mobilization exercises involving cervical lateral bending (chin-to-girth, chin-to-hip, and chin-to-tarsus) showed increased lateral bending of the cervical and thoracolumbar intervertebral joints as the horse stretched further caudally [27].

Another exercise-based therapeutic approach used lightweight (55 g) tactile stimulators attached loosely around the hind pasterns to increase the range of joint motion, re-educate the movement, and strengthen the muscles of the hind limbs. Skin stimulation of the pastern and coronet was thought to mimic the effects of the tripping reflex. Speed and stride duration did not differ between conditions, but when stimulators were present, stance duration decreased, swing duration increased, and peak height of the hind hooves increased due to greater flexion of the stifle, tarsal, metatarsophalangeal, and distal interphalangeal joints during the swing phase [18]. A subsequent study [23] compared the effects of four types of pastern stimulators (10 g strap, 55 g tactile stimulators, 700 g weights, 700 g weights with tactile stimulators added) on trot kinematics. At the same trotting speed, stance duration was shorter and swing duration correspondingly longer with all stimulators except the strap. Peak hoof height was significantly higher with tactile stimulators and with weights, and there was a further significant increase in peak hoof height with the combined tactile-weighted stimulator. Individual horses varied in their responses, and the shape of the hoof flight arc differed between stimulators. Overall, the use of pastern stimulators increased swing phase flexions of the stifle, tarsal, and fetlock joints during trotting and can serve as a rehabilitation tool when flexion of these joints has been reduced, for example, by immobilization. It was recommended that the use of lightweight tactile stimulators should precede the use of weights.

A study was based on the use of acoustic myography [44] in eight horses in which the superficial gluteal muscle in the left hind limb was slightly but significantly weaker as determined by having a higher ESTi score when the horses circled to the left. (The ESTi score is a measurement specific to acoustic myography for assessing muscle function). Horses were trained every third day for one hour wearing a lightweight (82 g) bell boot on the left hind limb. After training with the bell boot for 6 weeks, acoustic myography showed that the asymmetry in the left hind limb on the left circle had decreased, but on the right circle an imbalance had developed that was thought to represent on over-compensation.

A randomized clinical trial assessed whether the contribution of dynamic mobilization exercises and gymnastic training improved the quality of the walk stride and epaxial muscle size in nine hippotherapy horses [31]. Horses that performed dynamic mobilization exercises (cervical flexion, extension, and lateral bending to both sides) showed a significant increase in cross-sectional area of multifidus muscle and a non-significant increase in thickness of longissimus dorsi. Horses that also performed gymnastic exercises (pelvic tilt, backing up, walking tight circles, stepping over a raised pole) showed increases in stride length and tracking distance at walk [31].

The same dynamic mobilization exercises, together with core strengthening exercises and balancing exercises, were proposed for use in horses with back pain in a narrative review [32]. This review also explored the benefits of a range of exercises at different gaits and speeds, on circles and gradients, jumping, poles, and unstable footing in rehabilitation of horses with back pain. When available, evidence-based research to support the use of specific exercises was included. 

A narrative review on rehabilitation assessment and interventions described exercise as one of the most frequently used interventions [33]. It described essentially the same dynamic mobilization, core training, and balancing exercises as the previous review [32] and also recommended exercise at different gaits and speeds, spiraling in and out on circles, changes of gait and speed, use of gradients to selectively load hind or forelimbs, jumping, poles, and unstable footing. 

A case study of a foal with tetanus included exercise as part of the treatment protocol, starting in weeks 3–4 [24]. During this phase, a walking frame was used to assist the foal with standing and walking and to allow longer periods of weight-bearing. In weeks 5–6, exercises were included to overcome residual problems, in particular the foal’s inability to raise and lower itself independently. Follow-up at 6 and 12 months did not reveal any deficiencies assessed by normal physical examination [24]. 

An observational study [26] evaluated the effect of athletic conditioning on degenerative suspensory ligament desmitis (DSLD) using six horses exercised on a treadmill for 30 min every other day at an average heart rate close to the anaerobic threshold (Table 1). The results showed that vertical impulse increased after 8 weeks of exercise and 4 months of pasture rest in DSLD-affected horses. The suspensory ligament fiber pattern subjectively improved with exercise in affected horses. Insulin levels significantly decreased from baseline in all horses after 4 and 8 weeks of exercise. The authors conclude that exercise did not seem to exacerbate and may have improved signs of DSLD in mild to moderate cases [26]. 

A case study that included exercise consisting of hand-walking and longing on a firm surface (5 to 20 min) in the rehabilitation of a radial fracture reported good results [28]. 

Davidson [34] presented a narrative review to describe controlled exercise protocols commonly used as part of the rehabilitation process to promote healing after muscle, bone, tendon, and ligament injury. Based on the author’s experience and some supporting literature, the following recommendations were made:

Muscle injury: week 1: stall rest; week 2: stall rest, walk 15 min; week 3: stall rest, walk 30 min; week 4: stall rest, walk 30 min, trot 5 min; week 5: stall rest, walk 20 min, trot 10 min; week 6: stall rest, walk 20 min, trot 20 min; week 7: stall rest, walk 20 min, trot 20 min, canter 5 min; week 8 onwards: small paddock turn out (6 × 6 m), gradually increase exercise to full training.

Bone injury: week 1–4: stall rest; week 5–6: stall rest, walk 15 min; week 7–8 (with radiographic evaluation of bone healing): stall rest, walk 30 min; week 9–16: small paddock turn out (6 × 6 m); week 16 onward: gradually increase exercise to full training.

Tendon/ligament injury: week 1–2: stall rest; week 3–4: stall rest, walk 5 min; week 5–6: stall rest, walk 10 min; week 7–8: stall rest, walk 15 min; week 9–10 (with lameness and ultrasound examination): stall rest, walk 20 min; week 11–12: stall rest, walk 25 min; weeks 13–14: stall rest, walk 30 min; weeks 15–16: stall rest, walk 35 min; week 17–18: stall rest, walk 40 min; weeks 19–20: stall rest, walk 40 min, trot 2 min; weeks 21–22: stall rest, walk 35 min, trot 5 min; weeks 23–24: stall rest, walk 30 min, trot 10 min; weeks 25–26: stall rest, walk 25 min, trot 15 min; weeks 27–28: stall rest, walk 20 min, trot 20 min; weeks 29–30: stall rest, walk 20 min, trot 20 min, canter 1 min; weeks 31–32: stall rest, walk 20 min, trot 20 min, canter 5 min; weeks 33–34: stall rest, walk 20 min, trot 20 min, canter 10 min; weeks 35–36: stall rest, walk 15 min, trot 20 min, canter 15 min; weeks 37–38: stall rest, walk 10 min, trot 20 min, canter 20 min; weeks 39–42: small paddock turn out (6 × 6 m), full flat work, no speed work or jumping; weeks 42 onward: small paddock turn out (6 × 6 m), full flat work, gradually introduce speed work or jumping [34]. 

Kaneps [3] described common rehabilitation approaches to surgical or medical equine conditions. In the exercise portion, he started with walk for 5 to 10 min once or twice a day with incremental increases based on observation of the horses’ soundness. Trot started only after 10–15 min hand walking for warm-up and was initially for short periods of 1–1.5 min. This author emphasized the need for exercise to be oriented toward the needs of the horse’s usual activity. 

In a retrospective study with 150 horses, exercise was, in some cases, part of the protocol used to test the effectiveness of high-power laser therapy [46]. The exercise program progressed as follows: week 1–2: walk 20 min on hard surface; week 3–6: walk 20 min on soft surface; week 7–10: walk 20 min on soft surface and introduce trotting increasing by 2 min per week; week 11–15: minimum 20 min walk, trot increasing 2 min per week and canter increasing 2 min per week; week 15–18: walk minimum 20 min, trot and canter as in normal flat work, and with increasing dressage exercises or jumping up to full workload. Based on 129 horses, the median time to return to the previous performance level was 6 months 

In a 2018 survey, exercise was found to be used frequently as part of the rehabilitation process in the form of controlled hand walking in 97.3%, as therapeutic exercises in 84.3%, by means of stretching in 83.3%, using an automatic horse walker in 56.7%, by the application of the Pessoa^®®^ lunging system in 46.2%, using a land treadmill in 39.9%, and with an Equiband in 27.4% of cases [49]. 

Finally, a retrospective analysis of 62 horses that survived at least 12 months after colic surgery (11 treated, 51 controls) investigated whether a 4-week program of core abdominal rehabilitation exercises (CARE) hastened postoperative recovery and allowed a more rapid return to training [51]. The exercise program started 4 weeks after surgery with dynamic mobilization exercises (chin-to-chest, chin-to-girth), sternal, withers and thoracic, lumbar and lumbosacral lifting; and caudal tail shift. Dynamic mobilization exercises performed in lateral bending and chin-between-fetlocks were added in week 2 with gradual increases in the number of repetitions of the exercises that were maintained through weeks 3–4. The CARE horses returned to work under saddle faster (median 60 days) compared with control horses (median 90 days) and were more likely to compete in some form of sport post-surgically (10/11 CARE, 24/51 controls). All CARE horses completed the program without complications, and they returned to work and training within a significantly shorter time than controls. It was concluded that core abdominal rehabilitation exercises could be safely performed after colic surgery and appeared to facilitate a faster recovery and return to work.

## 4. Conclusions

Overall, there is a lack of randomized clinical trials using large samples that can help describe evidence related to the different approaches cited. The large representation of narrative reviews and observational/descriptive studies, mostly based on the personal experience of the authors or citing the same results of the few studies conducted, needs to be supplemented by rigorously conducted, evidence-based research. Exercise, physical agents, and hydrotherapy appear to be the most commonly used options, but much of the information regarding their potential efficacy is based largely on the results of human studies. Some studies present options and parameterizations that can be useful for equine clinical practice, but it is clear that more evidence is needed with regard to parameters for use and efficacy of different rehabilitation methods in horses. 

## Figures and Tables

**Table 1:** Papers included in the review listed in chronological order.

Authors and Date	Type of Study	Type of Intervention and Comparator	Aims	Methods
[6] Bromiley, 1999	Narrative review	N/A	Generic description of equine physical therapy and associated interventions	Literature review and expert opinion
[7] Ridgway and Harman, 1999	Narrative review	N/A	Review interventions commonly used to treat equine back problems	Literature review and expert opinion
[8] Blackford et al., 2000	Pre-test post-test clinical trial	Iontophoretic drug delivery vs. intraarticular injection or passive delivery	Compare iontophoretic delivery of dexamethasone-phosphate with intraarticular injection or passive delivery	One group received a single intraarticular injection of 4 mL dexamethasone-phosphate (6 mg/mL). The second group received iontophoretic administration of dexamethasone-phosphate (6 mg/mL) at 4 mA for 40 min in the treated limb and at 0 mA for 40 min in the contralateral (control) limb. Blood and synovial fluid collected for drug screening.
[9] Arnold et al., 2003	Retrospective descriptive study. Multiple cases	Conservative management	Assess the effects of conservative management in non-articular fractures of the tibial tuberosity	Medical records analysis indicated conservative management consisting of box rest for 2 or 3 months with hand-walking starting in the 2nd month and increasing gradually (no amount indicated). Small paddock turnout or walking under saddle (depending on temperament) separately or combined until sound at slow trot followed by gradual integration into training program.
[10] Crowe et al., 2004	Descriptive study	Radial pressure wave therapy	Assess the use of radial pressure wave therapy in the treatment of chronic or recurrent proximal suspensory desmitis	Based on manufacturer’s recommendation, 3 treatments (starting on diagnosis, repeated at 2-week interval), 1000 impulses radial pressure waves on each side of limb, 10 Hz frequency. Treatments were followed by a controlled exercise program consisting of box rest with daily controlled walking exercise for 6 weeks gradually increasing from 3 to 60 min, followed by starting to trot at week 10–12 with a gradual increase in duration.
[11] Porter, 2005	Narrative review	Rehabilitation therapy description	Describe rehabilitation therapies for joint disease	Literature review and expert opinion
[12] Buchner and Schildboeck, 2006	Narrative review	Physical therapy	Describe physiotherapeutic methods applied to horses	Literature review and expert opinion
[13] Goff, 2009	Narrative review	Manual therapy	Describe manual therapies used in horses	Literature review and expert opinion
[14] Haussler, 2009	Narrative review	Manual therapy	Describe manual therapies used in horses	Literature review and expert opinion
[15] Paulekas and Haussler, 2009	Narrative review	Therapeutic exercise	Describe therapeutic exercises used for equine rehabilitation	Literature review and expert opinion
[16] Scott and Swenson, 2009	Narrative review	Massage therapy	Describe evidence for the benefits of massage therapy in equine rehabilitation	Literature review and expert opinion
[17] Clayton et al., 2010	Descriptive cross-sectional study	Dynamic mobilization in cervical flexion	Assess the influence on intravertebral angulation of 3 dynamic mobilization exercises proposed to be part of a rehabilitation protocol for neck and back pain	Three exercises in different positions of neck flexion: chin to chest, chin between carpi, chin between fore fetlocks
[18] Clayton et al., 2010	Descriptive cross-sectional study	Lightweight tactile limb stimulator	Measure swing phase kinematic and kinetic effects of tactile stimulation of the hind pastern and coronet	Lightweight (55 g) tactile stimulators attached to hind pasterns during trotting
[19] Frick, 2010	Narrative review	Stretching exercises	Review the use of stretching exercises to improve range of motion, prevent injury, decrease pain and offer indications/protocols for stretching exercises	Literature review and expert opinion
[20] Haussler, 2010	Narrative review	Manual therapies	Review manual therapies used in equine pain management	Literature review and expert opinion
[21] Haussler et al., 2010	Randomized clinical trial	Spinal manipulation and spinal mobilization	Determine whether a combination of spinal manipulative therapy and spinal mobilization is more effective in decreasing vertebral stiffness compared to spinal mobilization alone	Both groups received spinal mobilization applied rhythmically in the standing horse at 5 intervertebral sites (T14–T15, T17–T18, L1–L2, L3–L4, L6–S1). The spinal manipulation treatment group also received high-velocity, low-amplitude thrusts directed at the same 5 intervertebral sites. Weekly measurements pre- and post-intervention of spinal mobility, displacement amplitudes of the trunk, spinal stiffness, and trunk oscillation frequency.
[22] Hill and Crook, 2010	Descriptive, comparative crossover design	Massage therapy	Determine the effect of massage on equine hind limb protraction in terms of increment of passive and active hind limb protraction	The same physical therapist massaged (effleurage interspersed with 3 × 30 s bursts of circular kneading) the superficial gluteal, semitendinosus, biceps femoris, and semimembranosus muscles for 30 min in the treatment group. The control group received a sham massage procedure. After a “washout” period of 7 days, the treatment groups were reversed.
[23] Clayton et al., 2011	Cross-sectional study	Hind limb stimulation devices	Compare effects of different tactile stimulators on range of joint motion, re-education of movement patterns, and hind limb muscle strength	Optometric motion analysis of limb kinematics at trot using 4 types of stimulators: lightweight strap (10 g), tactile stimulator (55 g), flexible human wrist weights (700 g), and tactile stimulators added to limb weights (700 g).
[24] Mykkänen et al., 2011	Case study	Physical therapy-based intervention	Describe effects of a physical therapy program in a case of severe generalized tetanus	Physical therapy techniques adapted from those used for upper motor neuron syndromes, including passive range of motion exercises, assisted active range of motion exercises, stretching, massage and modified proprioceptive neuromuscular facilitation performed for 6-weeks. After 2 weeks, a walking frame was introduced to assist the foal to stand and walk, and to allow longer periods of weight-bearing. Two weeks later exercises introduced to overcome residual problems, in particular the foal’s inability to raise and lower itself independently and mild foot deformities treated with corrective shoeing for one month.
[25] Stubbs et al., 2011	Observational study	Dynamic mobilization exercises	Evaluate multifidus cross-sectional area before and after performing dynamic mobilization exercises for 12 weeks	Horses performed 3 cervical flexion exercises, one cervical extension exercise, and 3 cervical lateral bending exercises to both sides, 5 repetitions/exercise, 5 days/week for 12 weeks. Cross-sectional area of multifidus measured ultrasonographically on left and right sides at 5 thoracolumbar levels.
[26] Xie et al., 2011	Observational study	Exercise (athletic conditioning)	Describe the effect of athletic conditioning on degenerative suspensory ligament desmitis	Horses exercised on a treadmill for 30 min every other day for 8 weeks. Treadmill speed adjusted to maintain average heart rate around 170 beats/min (80% of maximum heart rate and close to anaerobic threshold) for 20 min. After 8 weeks of exercise, horses turned out on pasture for 4 months. Effects evaluated using gait analysis, radiology, ultrasonography, and measurements of serum insulin and glucose at start of study, after 8 weeks exercise, and after 4 months rest.
[27] Clayton et al., 2012	Observational study	Back problems, core training	Identify differences in cervical, thoracic, and lumbar intersegmental bending angles during 3 dynamic mobilization exercises in cervical lateral bending	Optometric motion analysis of skin-fixed markers on the head, cervical transverse processes (C1–C6), and dorsal spinous processes (T6, T8, T10, T16, L6, S2, and S4). Measures at rest and performing 3 dynamic cervical lateral bending exercises (chin-to-girth, chin-to-hip, and chin-to-tarsus) to the left and right sides.
[28] Carstanjen et al., 2012	Case study	Rehabilitation concept	Describe intervention for repair and rehabilitation of a radial fracture	Manual passive physiological mobilization started 8 weeks post-surgery. At 16 weeks after surgery, swim training (100 to 500 m), hand-walking, and lunging-exercise on a firm surface were included for 5 to 20 min.
[29] King et al., 2013	Narrative review	Aquatic therapy	Describe mechanisms of action of aquatic therapy in people, dogs, and horses and the potential value for treating equine osteoarthritis	Literature review and expert opinion
[30] Bierman et al., 2014	Randomized double-blinded, placebo-controlled, clinical trial	Pulsed electromagnetic field therapy	Describe the effect of pulsed electromagnetic field therapy on back pain	Pulsed magnetic field therapy applied using blankets for 40 min/day, intensity approx. 50 microtesla, rectangular impulse and variable frequency of 1–30 Hz. Placebo blanket for control.
[31] Oliveira et al., 2015	Randomized clinical trial	Gymnastic training and dynamic mobilization exercises	Assess effects of dynamic mobilization exercises and gymnastic training on quality of the walk stride and epaxial muscle size	Horses divided into 3 groups of 3 horses. All 3 groups performed hippotherapy sessions 3 days/week. Groups 2 and 3 performed dynamic mobilization exercises (3 cervical flexion exercises, one cervical extension exercise, and 3 cervical lateral bending exercises to left and right sides), 5 repetitions/session, 3 days/week. Group 3 also performed gymnastic exercises to strengthen abdominal and pelvic stabilization muscles: pelvic tilting (5 trials of 5 s duration), 10 steps rein back, walking around tight turns (3 circles to left and right), and walking over a 40 cm pole for 10 min. Evaluated using gait analysis and ultrasonographic measurements of size of multifidus and longissimus.
[32] Clayton, 2016	Narrative review	Core training	Describe techniques associated with core training and rehabilitation of horses with back pain	Literature review and expert opinion
[33] Daglish and Mama, 2016	Narrative review	General rehabilitation procedures	Describe pain assessment methods and interventions	Literature review and expert opinion
[34] Davidson, 2016	Narrative review	Controlled exercise	Describe the usage of controlled exercise and commonly used protocols	Literature review and expert opinion with general recommendations for injuries of specific tissues.
[35] Haussler, 2016	Narrative review	Joint mobilization and manipulation	Review joint mobilization and manipulation in the musculoskeletal management of the equine athlete	Literature review and expert opinion
[3] Kaneps, 2016	Narrative review	General rehabilitation procedures	Review common rehabilitation approaches to surgical or medical equine conditions	Literature review and expert opinion
[36] Schlachter and Lewis, 2016	Narrative review	Electrophysical modalities	Describe common electrophysical therapies used for equine athletes	Literature review and expert opinion
[37] Guedes, 2017	Narrative review	Pain management	Review the pain mechanism and describe pain management in equine rehabilitation	Literature review and expert opinion
[38] Halsberghe, 2017	Observational, pilot study	Whole body vibration	Verify long-term and immediate effects of whole-body vibration on chronic lameness	Lameness diagnosed by clinical assessment (AAEP lameness scale). All horses stood on a vibration platform during 30 min sessions at 40 Hz, amplitude 0.8 mm, acceleration 4.9 m/s^2^, 5 days/week for 60 days. Normal exercise routine maintained. Kinematic data acquired by inertial sensors.
[39] King et al., 2017	Randomized clinical trial	Underwater treadmill	16, 2- to 5-year-old horses with surgically induced osteoarthritis in one carpal joint	One week after surgery, horses randomly assigned to treatment or control groups. Both groups exercised at walk on the treadmill, but water added only for the treatment group. Effects evaluated by lameness examinations, and kinematic, kinetic, and electromyographic analyses during overground locomotion, diagnostic imaging, goniometry, and diagnostic imaging.
[40] Mattos et al., 2017	Randomized clinical trial	Therapeutic bandages	Verify the contribution of kinesiology taping to control post-operative swelling following arthroscopy of the tibio-patellofemoral joint	Kinesiology taping of the stifle using a FAN technique with 10% tension (original tension from factory). Tape applied 12–72 h post-surgery.
[41] Nankervis et al., 2017	Narrative review	Overground and water treadmills	Present evidence to inform the use of overground and water treadmills for rehabilitation of injury	Literature review and expert opinion
[42] Contino, 2018	Narrative review	General rehabilitation procedures	Describe the common management and rehabilitation of joint injury in sports horses	Literature review and expert opinion
[43] Gutierrrez-Nibeyro et al., 2018	Narrative review	Conservative management	Describe conservative and surgical treatment options for common equine foot problems	Literature review and expert opinion
[44] Jensen et al., 2018	Clinical study	Proprioceptive stimulation	Describes the effects of using a bell boot on a hind limb with imbalanced function of the superficial gluteal muscle	Eight trained horses that were shown by acoustic myography to have an imbalance of superficial gluteal muscle function when circling to the left. Horses were trained for 6 weeks with a single bell boot applied to the “weaker” hind limb. Evaluation based on improved acoustic myography scores.
[45] Nowlin et al., 2018	Pair-matched control study	Vibration therapy	Verify the effects of vibration therapy on performance improvement and healing in competitive horses	Treatment group: vibration platform at 50 Hz for 30 min. Control group: 30 min on the platform while turned off.
[46] Pluim et al., 2018	Retrospective, observational clinical study	High-power laser therapy	Verify the effectiveness of high-power laser therapy in treatment of 150 sports horses diagnosed ultrasonographically with tendinopathy or desmopathy	Horses treated once daily for 20 min using the manufacturer’s pre-established protocol to provide approximately 250 J/cm^3^ per treatment to injured area for 2 weeks. Additional pharmacological and physical treatments applied based on clinical judgement. Diagnostic ultrasonography used at start of study, at completion of laser treatment, and 4 weeks after treatment ceased. A progressive exercise rehabilitation protocol was used.
[47] Proctor-Brown et al., 2018	Retrospective study	Cryotherapy	Verify the benefits of digital cryotherapy on distal limb conditions	Sleeve-style digital cryotherapy. Three administration protocols: continuous (211 cases), interrupted (57 cases), and intermittent (17 cases).
[48] Tranquille et al., 2018	Survey	Water treadmill	To understand how and why the water treadmill is used in equine rehabilitation	n/a
[49] Wilson et al., 2018	Survey	Rehabilitation modalities	Describe rehabilitation modalities used the horse	n/a
[50] Carrozzo et al., 2019	Retrospective description	Non-invasive low frequency ultrasound	Describe the effect of the therapeutic ultrasound in the treatment of lameness	Low frequency therapeutic ultrasound started at 50% dosage for each protocol for 1 min for horse habituation to stimulus, then increased to desired dosage. Protocols defined by injury stage (acute, subacute, chronic), using different transducers for pulsed and continuous emission, and various shapes for different wave emission.
[51] Holcombe et al., 2019	Retrospective cohort	Core training	Determine effects of 4-week core abdominal rehabilitation exercises (CARE) after colic surgery in terms of safety and return to training	The CARE exercise program is a 4-week protocol, based on increasing the type and number of core training exercises on a weekly basis.
[52] Muñoz et al., 2019	Narrative review	Water treadmill	Review the principles of aquatic therapy, types of aquatic exercise, and rehabilitation parameters for specific injuries	Describes pros and cons of the use of a water treadmill exercise in a rehabilitation program for a variety of injuries.
[53] Trager et al., 2019	Observational study	Extracorporeal shockwave therapy	Assess effects of extracorporeal shockwave therapy on mechanical nociceptive threshold and cross-sectional area of multifidus muscle	Shockwave therapy used in 3 treatment sessions, 2 weeks apart (days 0, 14 and 28), 80 mm probe, power E4 (penetration depth 113 mm, energy flux density 0.13 mJ/mm^2^), 45° angulation of probe. Continuous movement of probe between T12 and L5 for 1500 pulses (750 each side).
